# A triclinic polymorph of tri­cyclo­hexyl­phosphane sulfide: crystal structure and Hirshfeld surface analysis

**DOI:** 10.1107/S205698901700353X

**Published:** 2017-03-10

**Authors:** Yi Jiun Tan, Chien Ing Yeo, Nathan R. Halcovitch, Mukesh M. Jotani, Edward R. T. Tiekink

**Affiliations:** a Research Centre for Crystalline Materials, School of Science and Technology, Sunway University, 47500 Bandar Sunway, Selangor Darul Ehsan, Malaysia; b Department of Chemistry, Lancaster University, Lancaster LA1 4YB, United Kingdom; c Department of Physics, Bhavan’s Sheth R. A. College of Science, Ahmedabad, Gujarat 380001, India

**Keywords:** crystal structure, triorganophosphane sulfide, polymorph, Hirshfeld surface analysis

## Abstract

The title compound, (C_6_H_11_)_3_PS (systematic name: tri­cyclo­hexyl-λ^5^-phosphane­thione), is a triclinic (*P*-1, *Z*′ = 1) polymorph of the previously reported ortho­rhom­bic form (*Pnma*, *Z*′ = 1/2) [Kerr *et al.* (1977 ▸). *Can. J. Chem*. **55**, 3081–3085; Reibenspies *et al.* (1996 ▸). *Z. Kristallogr*. **211**, 400]. While conformational differences exist between the non-symmetric mol­ecule in the triclinic polymorph, *cf*. the mirror-symmetric mol­ecule in the ortho­rhom­bic form, these differences are not chemically significant. The major feature of the mol­ecular packing in the triclinic polymorph is the formation of linear chains along the *a* axis sustained by methine-C—H⋯S(thione) inter­actions. The chains pack with no directional inter­actions between them. The analysis of the Hirshfeld surface for both polymorphs indicates a high degree of similarity, being dominated by H⋯H (*ca* 90%) and S⋯H/H⋯S contacts.

## Abstract 2

The conformation found for (C_6_H_11_)_3_P=S in the triclinic polymorph lacks the mirror symmetry found in the ortho­rhom­bic form. Nevertheless, the conformations are in essential agreement. In the crystal, linear supra­molecular chains are sustained by methine-C—H⋯S(thione) inter­actions.

## Chemical context   

Recent inter­est in the chemistry of phosphanegold(I) di­thio­carbamate compounds stems from their potential as anti-cancer agents (de Vos *et al.* 2004 ▸; Ronconi *et al.* 2005 ▸; Gandin *et al.* 2010 ▸; Jamaludin *et al.* 2013 ▸; Keter *et al.* 2014 ▸; Altaf *et al.* 2015 ▸). In keeping with the increasing inter­est in gold compounds as potential anti-microbial agents to meet the challenges of microbes developing resistance to available chemotherapies (Glišić & Djuran, 2014 ▸) and in recognition of the potential of metal di­thio­carbamates as anti-microbial agents (Hogarth, 2012 ▸), the anti-bacterial properties of phosphanegold(I) di­thio­carbamates have also been explored in recent times (Sim *et al.*, 2014 ▸; Chen *et al.*, 2016 ▸). For example, the ‘all-eth­yl’ compound, Et_3_PAu(S_2_CNEt_2_), exhibits broad-range activity against Gram-positive and Gram-negative bacteria and was shown to be bactericidal against methicillin-resistant *Staphylococcus aureus* (MRSA) (Chen *et al.*, 2016 ▸). As an extension of these studies, investigations into the anti-microbial potential of related bis­(phosphane)copper(I) di­thio­carbamates and their silver(I) analogues were undertaken, again revealing inter­esting results and dependency of activity upon phosphane- and di­thio­carbamate-bound substituents (Jamaludin *et al.*, 2016 ▸). During further investigations in this field, the title compound, Cy_3_P=S (I), was isolated as a decomposition product from a long-term (months) recrystallization of an acetone solution containing (Cy_3_P)_2_Ag(S_2_CNEt_2_). The crystal and mol­ecular structures of (I) are reported herein and the results compared with those of a previously determined ortho­rhom­bic polymorph, (II) (Kerr *et al.*, 1977 ▸; Reibenspies *et al.*, 1996 ▸). Further, a detailed comparison of the Hirshfeld surfaces for (I) and (II) is presented.
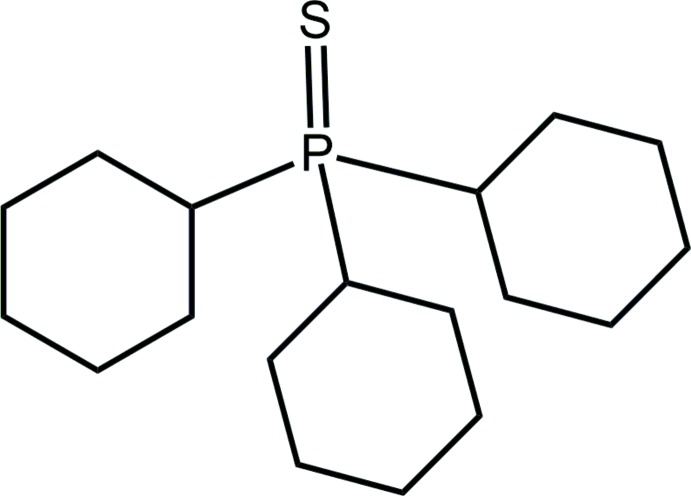


## Structural commentary   

**Figure 1:**
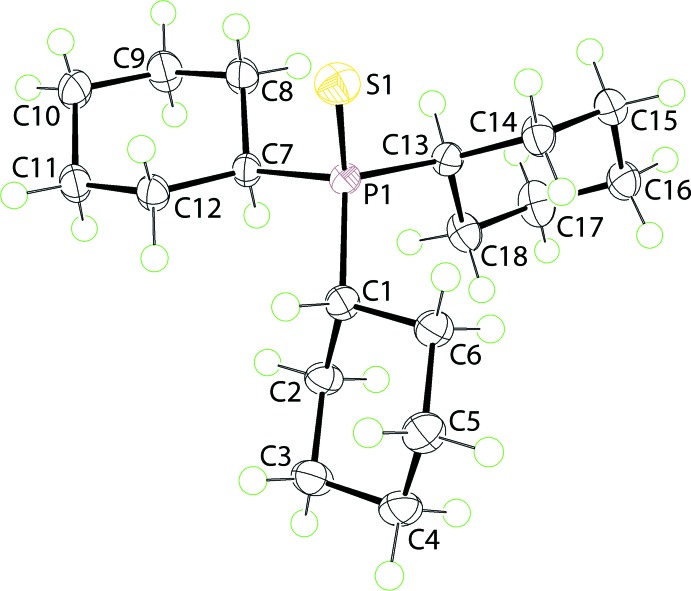
The mol­ecular structure of polymorph (I), showing the atom-labelling scheme and displacement ellipsoids at the 70% probability level.

The mol­ecular structure of (I), Fig. 1 ▸, features a tetra­hedrally coordinated P^V^ centre defined by a thione-S and three α-carbon atoms of the cyclo­hexyl substituents. The P1—C bond lengths span an experimentally distinct range of 1.8350 (14) to 1.8468 (15) Å, Table 1 ▸. The distortions from the ideal tetra­hedral geometry are relatively minor with the widest angles generally involving the thione-S atom. The cyclo­hexyl rings, each with a chair conformation, adopt orientations so that the methine-H atom is directed towards the thione-S atom in the cases of the C1- and C13-rings, *i.e*. are *syn*, with that of the C7-ring being *anti*.

As mentioned above, the structure of (I) has been reported previously in an ortho­rhom­bic form in two separate determinations (Kerr *et al.*, 1977 ▸; Reibenspies *et al.*, 1996 ▸). Data from the more recent determination, measured at 163 K (Reibenspies *et al.*, 1996 ▸), are included in Table 1 ▸. The major difference in (II) is that the mol­ecule lies on a crystallographic mirror plane; the 2 × *syn* plus 1 × *anti*-conformation of the methine-H atoms with respect to the thione-S atom persists. In (II), the P—C bond lengths are equal within experimental error. However, differences are apparent in the bond angles subtended at the P^V^ centre whereby the angles in (II) span a wider range, *i.e*. 8.5°, *cf*. 6.3 ° in (I). Also, the widest angle at the P1 atom in (II) is subtended by the symmetry-related cyclo­hexyl rings.

**Figure 2:**
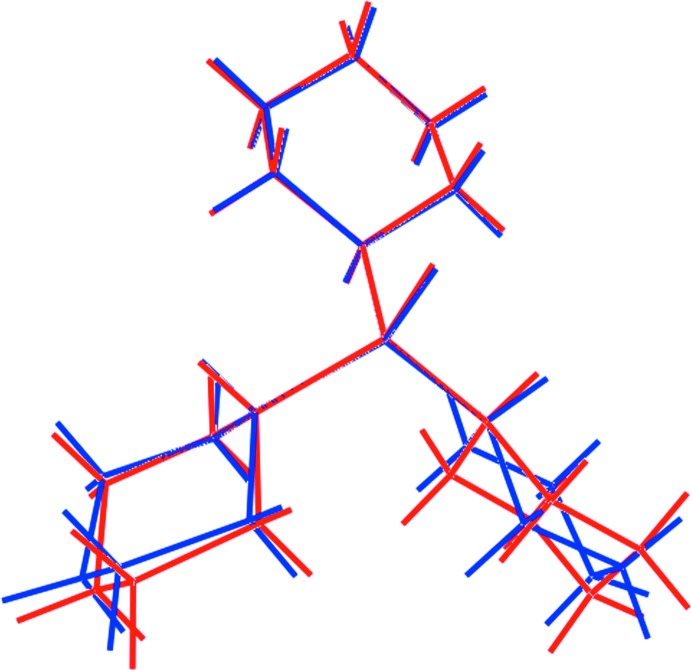
Overlay diagram of polymorphs (I), red image, and (II), blue image. The mol­ecules are overlapped so the three α-C atoms of the cyclo­hexyl rings are coincident.

An overlay diagram for (I) and (II) is shown in Fig. 2 ▸, which highlights the coincidence of the cyclo­hexyl ring associated with the methine-H atom having the *anti*-disposition with respect to the thione-S atom. Clearly, there are conformational differences apparent between the cyclo­hexyl rings related across the pseudo- and crystallographic mirror planes in (I) and (II), respectively.

## Supra­molecular features   

**Figure 3:**
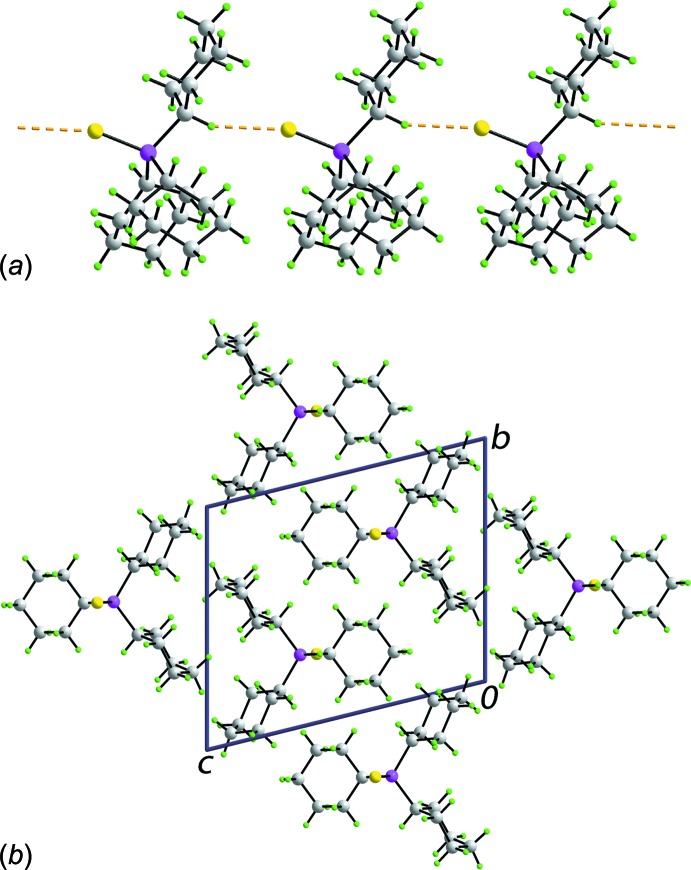
Mol­ecular packing in polymorph (I), showing (*a*) a linear supra­molecular chain mediated by methine-C—H⋯S(thione) inter­actions aligned along the *a* axis and (*b*) a view of the unit-cell contents in projection down the *a* axis. The C—H⋯S inter­actions are shown as orange dashed lines.

The only directional supra­molecular inter­actions in the crystal of (I) identified in *PLATON* (Spek, 2009 ▸) are methine-C—H7⋯S(thione) contacts, *i.e*. involving the *anti*-disposed thione-S and methine-H atoms, Table 2 ▸. These lead to a linear chain aligned along the *a* axis as illustrated in Fig. 3 ▸ *a*. The chains pack with no directional inter­actions between them, Fig. 3 ▸ *b*.

**Figure 4:**
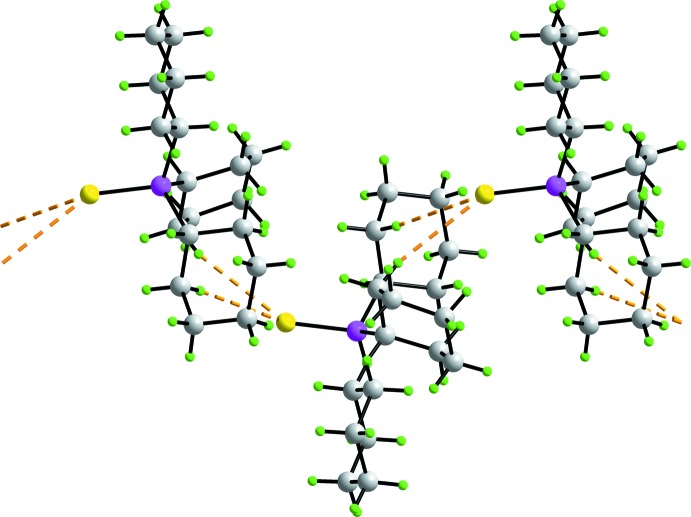
Mol­ecular packing in polymorph (II), showing a zigzag supra­molecular chain along the *a* axis mediated by methyl­ene-C—H⋯S(thione) inter­actions, shown as orange dashed lines.

In the original report of polymorph (II), it was stated ‘*There are no unusual inter-mol­ecular contacts’* (Kerr *et al.*, 1977 ▸); no comment on the mol­ecular packing was made in the redetermination (Reibenspies *et al.*, 1996 ▸). As seen from Fig. 4 ▸, supra­molecular zigzag chains are evident in the mol­ecular packing of (II), but these are sustained by weak methyl­ene-C—H⋯S(thione) inter­actions [H⋯S^i^ = 3.027 (2) Å, C⋯S^i^ = 3.938 (2) Å with the angle at H = 159° for (i) 1 + *x*, *y*, *z*] formed on either side of the mirror plane, so the sulfur atom forms two such contacts, and propagate along the *a* axis.

A more detailed analysis of the mol­ecular packing in (I) and (II) is given in *Hirshfeld surface analysis*.

## Hirshfeld surface analysis   

In order to gain more insight into the mol­ecular packing found in (I) and (II), the structures were subjected to a Hirshfeld surface analysis which was performed as described in a recent publication (Jotani *et al.*, 2016 ▸).

**Figure 5:**
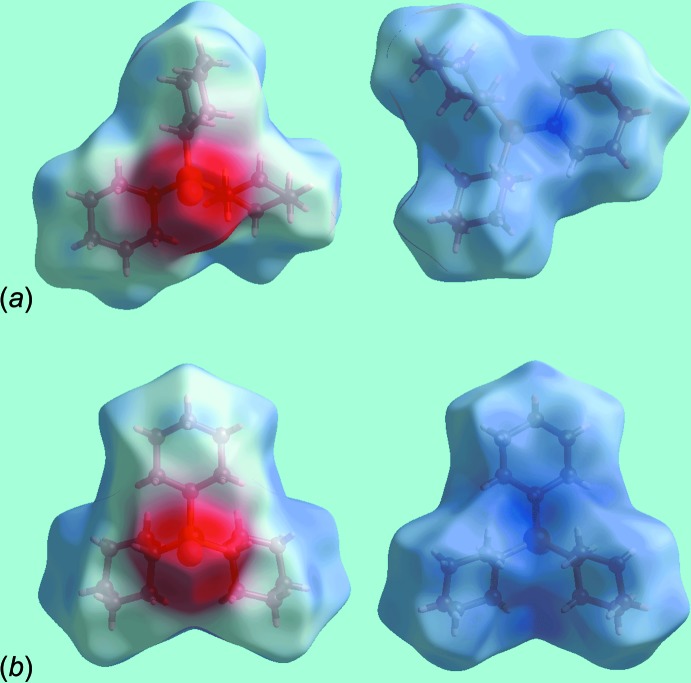
Views of the Hirshfeld surfaces for mapped over the electrostatic potential in the range ±0.075 au for (*a*) polymorph (I) and (*b*) polymorph (II).

**Figure 6:**
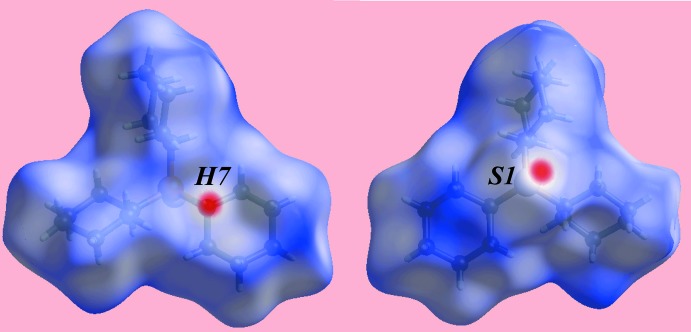
Views of the Hirshfeld surface for polymorph (I) mapped over *d*_norm_ over the range −0.160 to 1.823 au.

**Figure 7:**
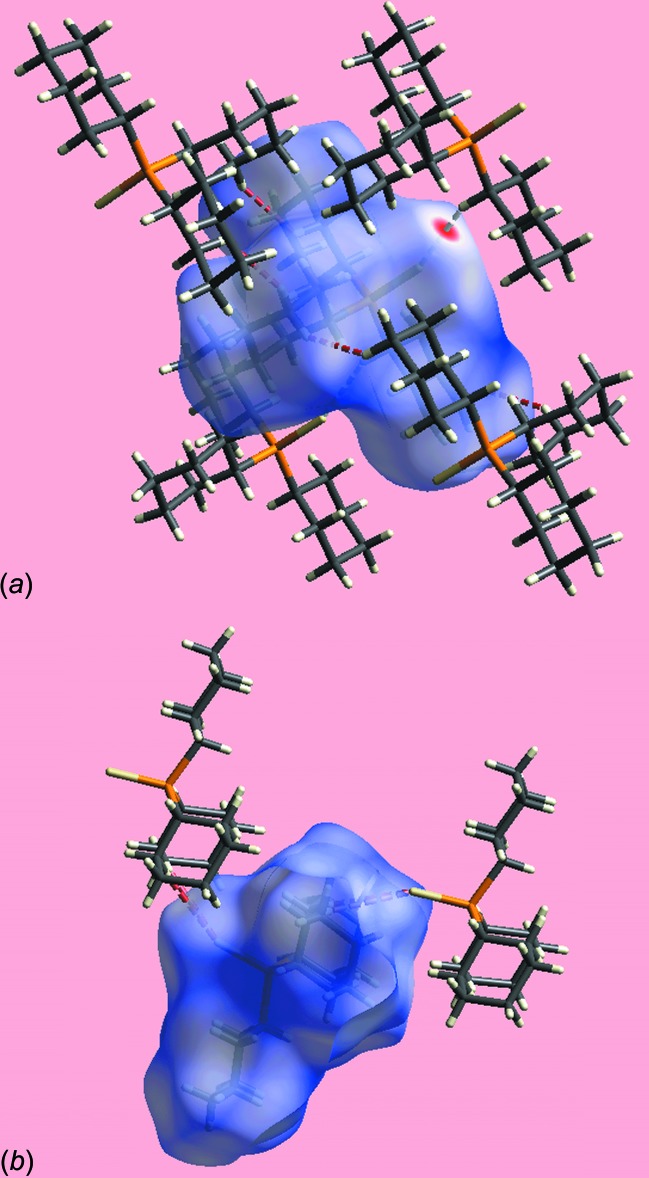
Views of the Hirshfeld surfaces mapped over *d*_norm_ about a reference mol­ecule highlighting inter­molecular C—H⋯S inter­actions and short inter­atomic H⋯H contacts as white and red dashed lines, respectively, for (*a*) polymorph (I) and (*b*) polymorph (II).

The different shapes of Hirshfeld surfaces mapped over electrostatic potential in Fig. 5 ▸ are indicative of the different mol­ecular conformations adopted by the cyclo­hexane rings in (I) and (II). A pair of bright-red spots appearing on the Hirshfeld surface mapped over *d*_norm_ near methine-H7 and thione-S1 for (I), Fig. 6 ▸, on the extremities of the mol­ecule represent the donor and acceptor of the C—H⋯S inter­action, Table 2 ▸. They are viewed as the respective blue (positive) and red (negative) regions on the Hirshfeld surface mapped over electrostatic potential, Fig. 5 ▸. The absence of characteristic spots on the *d*_norm_-mapped Hirshfeld surfaces in the ortho­rhom­bic polymorph (II) (not shown) indicates no similar inter­actions within the sum of the van der Waals radii; see below. The immediate environments about reference mol­ecules of (I) and (II) within the *d*_norm_-mapped Hirshfeld surfaces showing inter­molecular C—H⋯S inter­actions are displayed in Fig. 7 ▸ *a* and *b*, respectively. In the crystal of (II), the zigzag chain of weak inter­molecular methyl­ene-C—H⋯S(thione) contacts on either side of the crystallographic mirror plane is viewed as the pair of red dashed lines in Fig. 7 ▸ *b* (see above).

**Figure 8:**
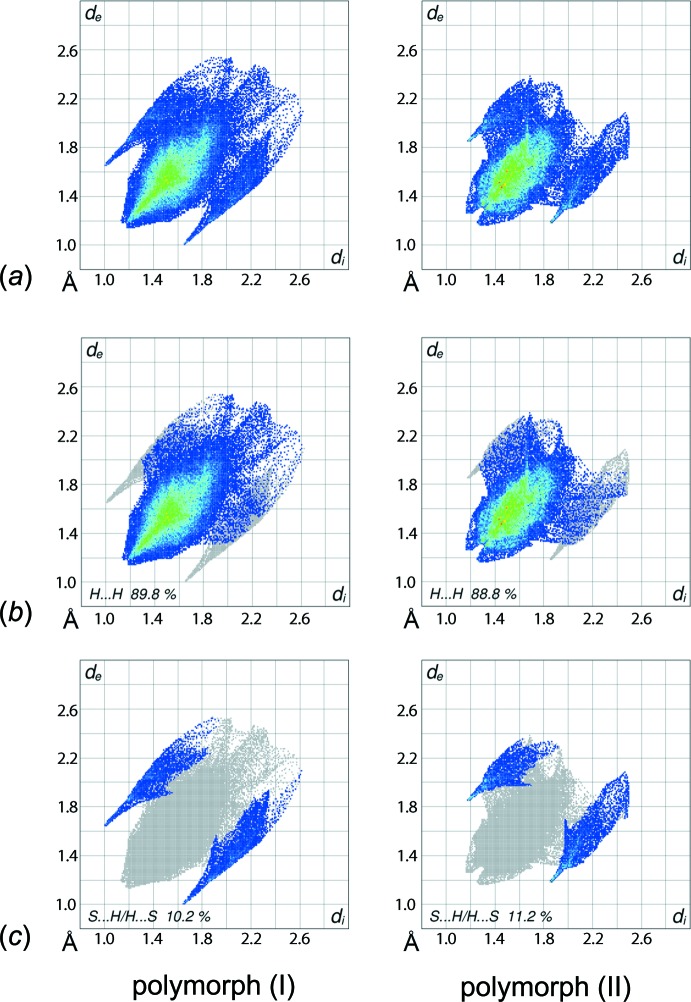
Fingerprint plots for polymorph (I) and polymorph (II), showing (*a*) overall and those delineated into (*b*) H⋯H and (*c*) S⋯H/H⋯S contacts.

The overall two-dimensional fingerprint plots for (I) and (II), and those delineated into H⋯H and S⋯H/H⋯S contacts (McKinnon *et al.*, 2007 ▸) are illustrated in Fig. 8 ▸. It is inter­esting to note that in both polymorphs only sulfur and hydrogen atoms lie on the periphery of the Hirshfeld surfaces and contribute to inter­atomic contacts such as they are; the percentage contributions are as qu­anti­fied in Table 3 ▸. The different relative orientations of the cyclo­hexane rings in the two forms are also evident through the distinct distribution of points in their respective two-dimensional fingerprint plots, Fig. 8 ▸ *a*. In particular for (II), Fig. 8 ▸ *a*, the top region, corresponding to donor inter­actions is stunted with respect to the lower, acceptor region. For (I), a pair of small peaks at *d*_e_ + *d*_i_ < 2.4 Å in the fingerprint plot delineated into H⋯H contacts, Fig. 8 ▸ *b*, show the contribution from short inter­atomic H⋯H contacts in the mol­ecular packing, Table 4 ▸. This contrasts the situation for (II), where the pair of peaks occur at *d*_e_ + *d*_i_ > 2.4 Å, *i.e*. at separations greater than the sum of van der Waals radii. The relative strength of the inter­molecular C—H⋯S inter­actions in (I) and (II) are characterized from the fingerprint plots delineated into S⋯H/H⋯S contacts, Fig. 8 ▸ *c*, through the pair of spikes at *d*_e_ + *d*_i_ ∼ 2.7 Å and *d*_e_ + *d*_i_ ∼ 3.1 Å, respectively. The asymmetric distribution of points in the fingerprint plot delineated into S⋯H/H⋯S contacts for (II) in Fig. 8 ▸ *c* is the result of the orientation of the cyclo­hexane rings with respect to the crystallographic mirror plane. The upper region, corresponding to donor H⋯S contacts, contributes 4.7% to the surface *cf*. 6.5% in the lower region, corresponding to S⋯H acceptor contacts.

The similarity in the mol­ecular packing of (I) and (II) is reflected in the similarity in the physiochemical data collated in Table 5 ▸ and calculated in *Crystal Explorer* (Wolff *et al.*, 2012 ▸) and *PLATON* (Spek, 2009 ▸). While it is noted the values are very close for (I) and (II) (Table 5 ▸), the volume of the mol­ecule in (I) is slightly greater than that in (II), as is the surface area. However, the mol­ecule in (II) is marginally more globular and reflecting the lack of directional inter­actions between mol­ecules, allowing a closer approach, the density is greater than in (I). Nevertheless, the packing efficiency is marginally greater in (I), probably reflecting the lack of symmetry in the mol­ecule *cf*. (I).

## Database survey   

There are a number of triorganophosphane sulfide structures in the crystallographic literature (Groom *et al.*, 2016 ▸) with those conforming to the general formula *R*_3_P=S being summarized here. Thus, structures have been described with fractional atomic coordinates, for example with *R* = Me (Tasker *et al.*, 2005 ▸), *i*Pr (Staples & Segal, 2001 ▸), *t*Bu (Steinberger *et al.*, 2001 ▸), Ph (Foces-Foces & Llamas-Saiz, 1998 ▸; monoclinic polymorph), Ph (Ziemer *et al.*, 2000 ▸; triclinic polymorph), 2-tolyl (Cameron & Dahlèn, 1975 ▸), 3-tolyl (Cameron *et al.*, 1978 ▸), 4-FPh (Barnes *et al.*, 2007 ▸), 2-(Me_2_NCH_2_)_3_Ph (Rotar *et al.*, 2010 ▸), 2,4,6-Me_3_Ph (Garland *et al.*, 2013 ▸) and 2,4,6-(OMe)_3_Ph (Finnen *et al.*, 1994 ▸). Selected geometric data for these structures along with those for (I) and (II) are collected in Table 6 ▸. The *R* = Me and *i*Pr mol­ecules have crystallographic mirror symmetry as for (II) whereas the *R* = *t*Bu compound has crystallographically imposed threefold symmetry. Two polymorphs have been found for *R* = Ph, and each of these features two independent mol­ecules in the asymmetric unit.

The longest P=S bond length, *i.e*. 1.9748 (13) Å, is found in sterically encumbered (2,4,6-Me3Ph)_3_P=S (Garland *et al.*, 2013 ▸). That steric effects are not the only factors influencing the magnitude of the P=S bond length is realized in the structure of Me_3_P=S, with small, electron-donating groups, which has the second longest P=S bond length across the series. The comments on the lack of definitive trends in the S—P—C and C—P—C bond angles made above for (I) and (II) hold true across the series although, generally, the former are wider than the latter. Inter­estingly, in the threefold symmetric *t*Bu_3_P=S structure, all angles are about 109°.

## Synthesis and crystallization   

The title compound (I) is an unexpected product from the *in situ* reaction of (Cy_3_P)_2_AgCl with Na[S_2_CNEt_2_] in a 2:1 ratio. The preparation was as follows: Cy_3_P (Sigma–Aldrich; 0.6 mmol, 0.196 g) dissolved in acetone (20 ml) was added to an acetone solution (20 ml) of AgCl (Sigma–Aldrich; 0.3 mmol, 0.05 g) at room temperature. Then, Na[S_2_CNEt_2_] (BDH, 0.3 mmol, 0.08 g) in acetone (20 ml) was added to the reaction mixture followed by stirring for 4 h. The resulting mixture was filtered, covered to exclude light and left for evaporation at room temperature. Colourless crystals were obtained after four months. Yield: 0.132 g (55%), m.p.: 437–440 K. IR (cm^−1^): ν(P=S) 624 (*s*).

## Refinement   

Crystal data, data collection and structure refinement details are summarized in Table 7 ▸. Carbon-bound H atoms were placed in calculated positions (C—H = 0.99–1.00 Å) and were included in the refinement in the riding-model approximation, with *U*_iso_(H) set to 1.2*U*_eq_(C).

## Supplementary Material

Crystal structure: contains datablock(s) I, global. DOI: 10.1107/S205698901700353X/hb7665sup1.cif

Structure factors: contains datablock(s) I. DOI: 10.1107/S205698901700353X/hb7665Isup2.hkl

Supporting information file. DOI: 10.1107/S205698901700353X/hb7665Isup3.cml

CCDC reference: 1536014

Additional supporting information:  crystallographic information; 3D view; checkCIF report

## Figures and Tables

**Table 1:** Geometric parameters (Å, °) for the triclinic (I) and ortho­rhom­bic (II) polymorphs of Cy_3_P=S

Parameter	triclinic polymorph	ortho­rhom­bic polymorph^*a*^
P1=S1	1.9548 (5)	1.9612 (11)
P1—C1	1.8435 (14)	1.842 (3)
P1—C7	1.8350 (14)	1.836 (2)
P1—C13	1.8468 (15)	1.836 (2)
S1—P1—C1	109.99 (5)	112.16 (11)
S1—P1—C7	112.11 (5)	110.15 (7)
S1—P1—C13	111.60 (5)	110.15 (7)
C1—P1—C7	105.82 (6)	105.22 (9)
C1—P1—C13	105.70 (6)	105.22 (9)
C7—P1—C13	111.43 (6)	113.80 (10)

Notes: (*a*) the mol­ecule has crystallographic mirror symmetry with the S1, P1 and C1 atoms lying on the plane.

**Table 2:** Hydrogen-bond geometry (Å, °)

*D*—H⋯*A*	*D*—H	H⋯*A*	*D*⋯*A*	*D*—H⋯*A*
C7—H7⋯S1^i^	1.00	2.65	3.5961 (14)	157

Symmetry code: (i) 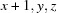.

**Table 3:** Percentage contributions of the different inter­molecular contacts to the Hirshfeld surface in (I) and (II)

Contact	% contribution in (I)	% contribution in (II)
H⋯H	89.8	88.8
S⋯H/H⋯S	10.2	11.2

**Table 4:** Short inter­atomic contacts in (I)

Contact	distance	symmetry operation
H6*A*⋯H15*B*	2.32	1 − *x*, 1 − *y*, 2 − *z*
H10*B*⋯H15*A*	2.37	1 − *x*, 1 − *y*, 1 − *z*

**Table 5:** Physiochemical properties for polymorphs (I) and (II)

Property	(I)	(II)
Volume, *V* (Å^3^)	436.83	430.96
Surface area, *A* (Å^2^)	351.03	345.83
*A*:*V* (Å^−1^)	0.804	0.802
Globularity, *G*	0.793	0.798
Asphericity, Ω	0.051	0.046
Density (g cm^−3^)	1.170	1.186
Packing index (%)	68.6	68.4

**Table 6:** Geometric parameters (Å, °) for selected *R*_3_P=S mol­ecules

R	P=S	S—P—C	C—P—C	Reference
Me^*a*^	1.9664 (7)	112.88 (6)–113.22 (8)	105.33 (8)–106.53 (8)	Tasker *et al.*, 2005 ▸
*i*Pr^*a*^	1.926 (3)	110.08 (19)–112.3 (2)	103.88 (19)–116.3 (4)	Staples *et al.*, 2001 ▸
*t*Bu^*b*^	1.9627 (15)	109.29 (14)	109.65 (19)	Steinberger *et al.*, 2001 ▸
Ph^*c*,*d*^	1.9554 (7)	112.16 (6)–113.47 (6)	103.70 (8)–107.76 (8)	Foces-Foces & Llamas-Saiz, 1998 ▸
	1.9547 (7)	112.28 (7)–113.67 (6)	103.12 (8)–107.53 (9)	
Ph^*d*,*e*^	1.9544 (9)	112.47 (7)–113.99 (7)	103.43 (8)–106.83 (8)	Ziemer *et al.*, 2000 ▸
	1.9529 (8)	111.97 (7)–113.19 (7)	103.61 (8)–107.38 (8)	
2-tol­yl^*d*^	1.953 (6)	110.7 (3)–114.2 (3)	101.4 (3)–110.6 (4)	Cameron & Dahlèn, 1975 ▸
	1.942 (5)	111.6 (2)–114.3 (2)	104.9 (3)–107.9 (3)	
3-tol­yl	1.937 (4)	112.1 (8)–112.6 (4)	105.5 (7)–108.2 (10)	Cameron *et al.*, 1978 ▸
4-FPh	1.9540 (9)	113.27 (8)–113.59 (8)	104.97 (10)–105.92 (10)	Barnes *et al.*, 2007 ▸
2,4,6-Me_3_Ph	1.9748 (13)	107.32 (11)–109.49 (12)	108.90 (16)–112.45 (15)	Garland *et al.*, 2013 ▸
2,4,6-(OMe)_3_Ph	1.9619 (12)	109.22 (11)–116.15 (11)	100.77 (14)–110.58 (14)	Finnen *et al.*, 1994 ▸
2-(Me_2_NCH_2_)_3_Ph	1.9622 (17)	110.66 (8)–116.15 (10)	103.51 (13)–106.33 (11)	Rotar *et al.*, 2010 ▸
Cy^*a*,*f*^	1.9612 (11)	110.15 (7)–112.16 (11)	105.22 (9)–113.80 (10)	Reibenspies *et al.*, 1996 ▸
Cy^*e*^	1.9548 (5)	109.99 (5)–112.11 (5)	105.70 (6)–111.43 (6)	this work

Notes: (*a*) The mol­ecule has crystallographic mirror symmetry with the S1, P1 and C1 atoms lying on the plane; (*b*) the mol­ecule has crystallographic threefold symmetry with the S1 and P1 atoms lying on the axis; (*c*) monoclinic polymorph; (*d*) two independent mol­ecules in the asymmetric unit; (*e*) triclinic polymorph; (*f*) ortho­rhom­bic polymorph.

**Table 7:** Experimental details

Crystal data	
Chemical formula	C_18_H_33_PS
*M*_r_	312.47
Crystal system, space group	Triclinic, *P* 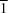
Temperature (K)	100
*a*, *b*, *c* (Å)	6.6400 (5), 10.8089 (9), 12.8818 (10)
α, β, γ (°)	103.430 (7), 98.467 (7), 91.912 (7)
*V* (Å^3^)	887.26 (12)
*Z*	2
Radiation type	Mo *K*α
μ (mm^−1^)	0.26
Crystal size (mm)	0.40 × 0.20 × 0.17

Data collection	
Diffractometer	Agilent SuperNova, Dual, Mo at zero, AtlasS2
Absorption correction	Multi-scan (*CrysAlis PRO*; Rigaku Oxford Diffraction, 2015 ▸)
*T*_min_, *T*_max_	0.926, 1.000
No. of measured, independent and observed [*I* > 2σ(*I*)] reflections	8658, 4208, 3739
*R*_int_	0.022
(sin θ/λ)_max_ (Å^−1^)	0.696

Refinement	
*R*[*F* ^2^ > 2σ(*F* ^2^)], *wR*(*F* ^2^), *S*	0.037, 0.097, 1.01
No. of reflections	4208
No. of parameters	181
H-atom treatment	H-atom parameters constrained
Δρ_max_, Δρ_min_ (e Å^−3^)	0.47, −0.35

Computer programs: *CrysAlis PRO* (Rigaku Oxford Diffraction, 2015 ▸), *SHELXS97* (Sheldrick, 2008 ▸), *SHELXL2014* (Sheldrick, 2015 ▸), *ORTEP-3 for Windows* (Farrugia, 2012 ▸), *QMol* (Gans & Shalloway, 2001 ▸), *DIAMOND* (Brandenburg, 2006 ▸) and *publCIF* (Westrip, 2010 ▸).

## supplementary crystallographic information

### Crystal data

**Table:**

C_18_H_33_PS	*Z* = 2
*M**_r_* = 312.47	*F*(000) = 344
Triclinic, *P*1	*D*_x_ = 1.170 Mg m^−^^3^
*a* = 6.6400 (5) Å	Mo *K*α radiation, λ = 0.71073 Å
*b* = 10.8089 (9) Å	Cell parameters from 4800 reflections
*c* = 12.8818 (10) Å	θ = 3.7–29.5°
α = 103.430 (7)°	µ = 0.26 mm^−^^1^
β = 98.467 (7)°	*T* = 100 K
γ = 91.912 (7)°	Prism, colourless
*V* = 887.26 (12) Å^3^	0.40 × 0.20 × 0.17 mm

### Data collection

**Table:**

Agilent SuperNova, Dual, Mo at zero, AtlasS2 diffractometer	4208 independent reflections
Radiation source: micro-focus sealed X-ray tube, SuperNova (Mo) X-ray Source	3739 reflections with *I* > 2σ(*I*)
Mirror monochromator	*R*_int_ = 0.022
ω scans	θ_max_ = 29.7°, θ_min_ = 2.8°
Absorption correction: multi-scan (CrysAlis PRO; Rigaku Oxford Diffraction, 2015)	*h* = −8→9
*T*_min_ = 0.926, *T*_max_ = 1.000	*k* = −13→12
8658 measured reflections	*l* = −16→17

### Refinement

**Table:**

Refinement on *F*^2^	0 restraints
Least-squares matrix: full	Hydrogen site location: inferred from neighbouring sites
*R*[*F*^2^ > 2σ(*F*^2^)] = 0.037	H-atom parameters constrained
*wR*(*F*^2^) = 0.097	*w* = 1/[σ^2^(*F*_o_^2^) + (0.0433*P*)^2^ + 0.4808*P*] where *P* = (*F*_o_^2^ + 2*F*_c_^2^)/3
*S* = 1.01	(Δ/σ)_max_ = 0.001
4208 reflections	Δρ_max_ = 0.47 e Å^−^^3^
181 parameters	Δρ_min_ = −0.35 e Å^−^^3^

### Special details

**Table:**

Geometry. All esds (except the esd in the dihedral angle between two l.s. planes) are estimated using the full covariance matrix. The cell esds are taken into account individually in the estimation of esds in distances, angles and torsion angles; correlations between esds in cell parameters are only used when they are defined by crystal symmetry. An approximate (isotropic) treatment of cell esds is used for estimating esds involving l.s. planes.

### Fractional atomic coordinates and isotropic or equivalent isotropic displacement parameters (Å^2^)

**Table:**

	*x*	*y*	*z*	*U*_iso_*/*U*_eq_	
S1	0.28983 (5)	0.28303 (4)	0.60522 (3)	0.02266 (11)	
P1	0.58024 (5)	0.29616 (3)	0.66467 (3)	0.01328 (10)	
C1	0.6361 (2)	0.17062 (13)	0.73806 (11)	0.0155 (3)	
H1	0.5711	0.0898	0.6877	0.019*	
C2	0.8619 (2)	0.14502 (14)	0.76697 (12)	0.0176 (3)	
H2A	0.9269	0.1300	0.7012	0.021*	
H2B	0.9347	0.2205	0.8197	0.021*	
C3	0.8780 (2)	0.02835 (15)	0.81546 (12)	0.0215 (3)	
H3A	0.8173	−0.0484	0.7599	0.026*	
H3B	1.0238	0.0157	0.8371	0.026*	
C4	0.7689 (2)	0.04367 (15)	0.91382 (12)	0.0215 (3)	
H4A	0.8396	0.1143	0.9727	0.026*	
H4B	0.7751	−0.0355	0.9400	0.026*	
C5	0.5457 (2)	0.07171 (15)	0.88609 (12)	0.0210 (3)	
H5A	0.4708	−0.0033	0.8337	0.025*	
H5B	0.4820	0.0873	0.9524	0.025*	
C6	0.5286 (2)	0.18808 (14)	0.83768 (12)	0.0183 (3)	
H6A	0.5910	0.2649	0.8927	0.022*	
H6B	0.3828	0.2012	0.8168	0.022*	
C7	0.7413 (2)	0.27103 (13)	0.55847 (11)	0.0151 (3)	
H7	0.8872	0.2799	0.5940	0.018*	
C8	0.7119 (2)	0.37069 (14)	0.49033 (12)	0.0209 (3)	
H8A	0.5663	0.3672	0.4581	0.025*	
H8B	0.7496	0.4570	0.5376	0.025*	
C9	0.8424 (3)	0.34686 (15)	0.40031 (12)	0.0252 (3)	
H9A	0.8170	0.4105	0.3565	0.030*	
H9B	0.9886	0.3571	0.4325	0.030*	
C10	0.7926 (3)	0.21275 (15)	0.32763 (12)	0.0254 (3)	
H10A	0.8810	0.1984	0.2710	0.030*	
H10B	0.6488	0.2042	0.2915	0.030*	
C11	0.8260 (2)	0.11291 (14)	0.39418 (12)	0.0203 (3)	
H11A	0.7889	0.0267	0.3465	0.024*	
H11B	0.9721	0.1172	0.4257	0.024*	
C12	0.6967 (2)	0.13517 (14)	0.48499 (11)	0.0178 (3)	
H12A	0.7261	0.0720	0.5290	0.021*	
H12B	0.5503	0.1224	0.4532	0.021*	
C13	0.6633 (2)	0.45629 (13)	0.75178 (11)	0.0155 (3)	
H13	0.6674	0.5136	0.7012	0.019*	
C14	0.5091 (2)	0.51104 (15)	0.82558 (12)	0.0202 (3)	
H14A	0.3716	0.5046	0.7820	0.024*	
H14B	0.5027	0.4609	0.8804	0.024*	
C15	0.5722 (2)	0.65042 (15)	0.88193 (12)	0.0219 (3)	
H15A	0.5689	0.7014	0.8271	0.026*	
H15B	0.4736	0.6838	0.9304	0.026*	
C16	0.7857 (2)	0.66490 (14)	0.94750 (12)	0.0205 (3)	
H16A	0.7863	0.6202	1.0064	0.025*	
H16B	0.8246	0.7563	0.9807	0.025*	
C17	0.9411 (2)	0.60985 (15)	0.87601 (13)	0.0242 (3)	
H17A	1.0768	0.6154	0.9212	0.029*	
H17B	0.9516	0.6609	0.8222	0.029*	
C18	0.8796 (2)	0.47037 (14)	0.81713 (12)	0.0200 (3)	
H18A	0.8846	0.4175	0.8706	0.024*	
H18B	0.9779	0.4392	0.7678	0.024*	

### Atomic displacement parameters (Å^2^)

**Table:**

	*U*^11^	*U*^22^	*U*^33^	*U*^12^	*U*^13^	*U*^23^
S1	0.01585 (19)	0.0258 (2)	0.0254 (2)	0.00077 (14)	0.00114 (14)	0.00571 (15)
P1	0.01333 (18)	0.01384 (18)	0.01275 (17)	0.00045 (12)	0.00201 (12)	0.00351 (13)
C1	0.0171 (7)	0.0152 (7)	0.0156 (6)	0.0008 (5)	0.0049 (5)	0.0050 (5)
C2	0.0175 (7)	0.0190 (7)	0.0197 (7)	0.0038 (5)	0.0062 (5)	0.0090 (6)
C3	0.0237 (8)	0.0200 (8)	0.0248 (8)	0.0066 (6)	0.0081 (6)	0.0101 (6)
C4	0.0263 (8)	0.0204 (8)	0.0222 (7)	0.0040 (6)	0.0074 (6)	0.0116 (6)
C5	0.0240 (8)	0.0209 (8)	0.0217 (7)	0.0008 (6)	0.0083 (6)	0.0095 (6)
C6	0.0197 (7)	0.0197 (7)	0.0189 (7)	0.0034 (5)	0.0074 (5)	0.0084 (6)
C7	0.0200 (7)	0.0129 (7)	0.0130 (6)	0.0013 (5)	0.0047 (5)	0.0026 (5)
C8	0.0334 (8)	0.0140 (7)	0.0174 (7)	0.0034 (6)	0.0080 (6)	0.0054 (5)
C9	0.0427 (10)	0.0172 (8)	0.0207 (7)	0.0040 (6)	0.0140 (7)	0.0089 (6)
C10	0.0429 (10)	0.0208 (8)	0.0152 (7)	0.0069 (7)	0.0094 (6)	0.0061 (6)
C11	0.0293 (8)	0.0151 (7)	0.0169 (7)	0.0029 (6)	0.0066 (6)	0.0026 (5)
C12	0.0249 (7)	0.0129 (7)	0.0165 (7)	0.0009 (5)	0.0058 (5)	0.0039 (5)
C13	0.0162 (7)	0.0158 (7)	0.0146 (6)	0.0008 (5)	0.0029 (5)	0.0034 (5)
C14	0.0166 (7)	0.0219 (8)	0.0194 (7)	0.0024 (5)	0.0032 (5)	−0.0006 (6)
C15	0.0252 (8)	0.0205 (8)	0.0185 (7)	0.0075 (6)	0.0025 (6)	0.0012 (6)
C16	0.0250 (8)	0.0150 (7)	0.0194 (7)	−0.0003 (5)	0.0024 (6)	0.0004 (5)
C17	0.0205 (8)	0.0203 (8)	0.0282 (8)	−0.0052 (6)	0.0047 (6)	−0.0011 (6)
C18	0.0164 (7)	0.0177 (7)	0.0233 (7)	−0.0001 (5)	0.0028 (6)	0.0003 (6)

### Geometric parameters (Å, º)

**Table:**

S1—P1	1.9548 (5)	C9—H9A	0.9900
P1—C1	1.8435 (14)	C9—H9B	0.9900
P1—C7	1.8350 (14)	C10—C11	1.529 (2)
P1—C13	1.8468 (15)	C10—H10A	0.9900
C1—C6	1.5356 (18)	C10—H10B	0.9900
C1—C2	1.5408 (19)	C11—C12	1.5302 (19)
C1—H1	1.0000	C11—H11A	0.9900
C2—C3	1.532 (2)	C11—H11B	0.9900
C2—H2A	0.9900	C12—H12A	0.9900
C2—H2B	0.9900	C12—H12B	0.9900
C3—C4	1.530 (2)	C13—C18	1.5376 (19)
C3—H3A	0.9900	C13—C14	1.5391 (19)
C3—H3B	0.9900	C13—H13	1.0000
C4—C5	1.530 (2)	C14—C15	1.527 (2)
C4—H4A	0.9900	C14—H14A	0.9900
C4—H4B	0.9900	C14—H14B	0.9900
C5—C6	1.529 (2)	C15—C16	1.523 (2)
C5—H5A	0.9900	C15—H15A	0.9900
C5—H5B	0.9900	C15—H15B	0.9900
C6—H6A	0.9900	C16—C17	1.527 (2)
C6—H6B	0.9900	C16—H16A	0.9900
C7—C8	1.540 (2)	C16—H16B	0.9900
C7—C12	1.5437 (19)	C17—C18	1.533 (2)
C7—H7	1.0000	C17—H17A	0.9900
C8—C9	1.528 (2)	C17—H17B	0.9900
C8—H8A	0.9900	C18—H18A	0.9900
C8—H8B	0.9900	C18—H18B	0.9900
C9—C10	1.528 (2)		
			
C7—P1—C1	105.82 (6)	C10—C9—H9B	109.5
C7—P1—C13	105.70 (6)	C8—C9—H9B	109.5
C1—P1—C13	111.43 (6)	H9A—C9—H9B	108.1
C7—P1—S1	112.11 (5)	C9—C10—C11	110.38 (12)
C1—P1—S1	109.99 (5)	C9—C10—H10A	109.6
C13—P1—S1	111.60 (5)	C11—C10—H10A	109.6
C6—C1—C2	110.75 (11)	C9—C10—H10B	109.6
C6—C1—P1	111.78 (10)	C11—C10—H10B	109.6
C2—C1—P1	117.32 (10)	H10A—C10—H10B	108.1
C6—C1—H1	105.3	C12—C11—C10	110.93 (12)
C2—C1—H1	105.3	C12—C11—H11A	109.5
P1—C1—H1	105.3	C10—C11—H11A	109.5
C3—C2—C1	110.13 (12)	C12—C11—H11B	109.5
C3—C2—H2A	109.6	C10—C11—H11B	109.5
C1—C2—H2A	109.6	H11A—C11—H11B	108.0
C3—C2—H2B	109.6	C11—C12—C7	111.43 (12)
C1—C2—H2B	109.6	C11—C12—H12A	109.3
H2A—C2—H2B	108.1	C7—C12—H12A	109.3
C4—C3—C2	111.72 (12)	C11—C12—H12B	109.3
C4—C3—H3A	109.3	C7—C12—H12B	109.3
C2—C3—H3A	109.3	H12A—C12—H12B	108.0
C4—C3—H3B	109.3	C18—C13—C14	110.45 (11)
C2—C3—H3B	109.3	C18—C13—P1	115.68 (10)
H3A—C3—H3B	107.9	C14—C13—P1	113.42 (10)
C3—C4—C5	111.30 (12)	C18—C13—H13	105.4
C3—C4—H4A	109.4	C14—C13—H13	105.4
C5—C4—H4A	109.4	P1—C13—H13	105.4
C3—C4—H4B	109.4	C15—C14—C13	110.25 (12)
C5—C4—H4B	109.4	C15—C14—H14A	109.6
H4A—C4—H4B	108.0	C13—C14—H14A	109.6
C6—C5—C4	111.10 (12)	C15—C14—H14B	109.6
C6—C5—H5A	109.4	C13—C14—H14B	109.6
C4—C5—H5A	109.4	H14A—C14—H14B	108.1
C6—C5—H5B	109.4	C16—C15—C14	111.29 (12)
C4—C5—H5B	109.4	C16—C15—H15A	109.4
H5A—C5—H5B	108.0	C14—C15—H15A	109.4
C5—C6—C1	111.04 (12)	C16—C15—H15B	109.4
C5—C6—H6A	109.4	C14—C15—H15B	109.4
C1—C6—H6A	109.4	H15A—C15—H15B	108.0
C5—C6—H6B	109.4	C15—C16—C17	110.86 (12)
C1—C6—H6B	109.4	C15—C16—H16A	109.5
H6A—C6—H6B	108.0	C17—C16—H16A	109.5
C8—C7—C12	110.18 (11)	C15—C16—H16B	109.5
C8—C7—P1	111.69 (10)	C17—C16—H16B	109.5
C12—C7—P1	110.46 (10)	H16A—C16—H16B	108.1
C8—C7—H7	108.1	C16—C17—C18	111.30 (12)
C12—C7—H7	108.1	C16—C17—H17A	109.4
P1—C7—H7	108.1	C18—C17—H17A	109.4
C9—C8—C7	111.28 (12)	C16—C17—H17B	109.4
C9—C8—H8A	109.4	C18—C17—H17B	109.4
C7—C8—H8A	109.4	H17A—C17—H17B	108.0
C9—C8—H8B	109.4	C17—C18—C13	111.07 (12)
C7—C8—H8B	109.4	C17—C18—H18A	109.4
H8A—C8—H8B	108.0	C13—C18—H18A	109.4
C10—C9—C8	110.78 (13)	C17—C18—H18B	109.4
C10—C9—H9A	109.5	C13—C18—H18B	109.4
C8—C9—H9A	109.5	H18A—C18—H18B	108.0
			
C7—P1—C1—C6	173.93 (10)	P1—C7—C8—C9	−178.51 (10)
C13—P1—C1—C6	59.51 (11)	C7—C8—C9—C10	57.32 (17)
S1—P1—C1—C6	−64.79 (10)	C8—C9—C10—C11	−57.82 (18)
C7—P1—C1—C2	44.45 (12)	C9—C10—C11—C12	57.32 (17)
C13—P1—C1—C2	−69.97 (12)	C10—C11—C12—C7	−56.28 (16)
S1—P1—C1—C2	165.73 (9)	C8—C7—C12—C11	54.86 (16)
C6—C1—C2—C3	56.61 (16)	P1—C7—C12—C11	178.74 (10)
P1—C1—C2—C3	−173.43 (10)	C7—P1—C13—C18	−67.43 (12)
C1—C2—C3—C4	−56.03 (16)	C1—P1—C13—C18	47.06 (12)
C2—C3—C4—C5	55.46 (17)	S1—P1—C13—C18	170.45 (9)
C3—C4—C5—C6	−54.99 (17)	C7—P1—C13—C14	163.46 (10)
C4—C5—C6—C1	55.98 (16)	C1—P1—C13—C14	−82.05 (11)
C2—C1—C6—C5	−57.02 (16)	S1—P1—C13—C14	41.34 (11)
P1—C1—C6—C5	170.15 (10)	C18—C13—C14—C15	56.93 (16)
C1—P1—C7—C8	−179.57 (10)	P1—C13—C14—C15	−171.34 (10)
C13—P1—C7—C8	−61.26 (11)	C13—C14—C15—C16	−57.70 (16)
S1—P1—C7—C8	60.53 (11)	C14—C15—C16—C17	56.93 (17)
C1—P1—C7—C12	57.43 (11)	C15—C16—C17—C18	−55.49 (18)
C13—P1—C7—C12	175.73 (9)	C16—C17—C18—C13	55.34 (17)
S1—P1—C7—C12	−62.47 (10)	C14—C13—C18—C17	−55.97 (16)
C12—C7—C8—C9	−55.35 (16)	P1—C13—C18—C17	173.48 (10)

### Hydrogen-bond geometry (Å, º)

**Table:**

*D*—H···*A*	*D*—H	H···*A*	*D*···*A*	*D*—H···*A*
C7—H7···S1^i^	1.00	2.65	3.5961 (14)	157

Symmetry code: (i) *x*+1, *y*, *z*.
